# A multi-center, randomized, 12-month, parallel-group, feasibility study to assess the acceptability and preliminary impact of family navigation plus usual care versus usual care on attrition in managing pediatric obesity: a study protocol

**DOI:** 10.1186/s40814-023-01246-w

**Published:** 2023-01-23

**Authors:** Geoff D. C. Ball, Marcus G. O’Neill, Rafat Noor, Angela Alberga, Rima Azar, Annick Buchholz, Michelle Enright, Josie Geller, Josephine Ho, Nicholas L. Holt, Tracy Lebel, Rhonda J. Rosychuk, Jean-Eric Tarride, Ian Zenlea

**Affiliations:** 1 Department of Pediatrics, University of Alberta, 4-515 Edmonton Clinic Health Academy, 11405 87th Ave, Edmonton, AB T6G 1C9 Canada; 2 Department of Health, Kinesiology, and Applied Physiology, Concordia University, Montreal, QC Canada; 3 Psychobiology of Stress & Health Lab, Department of Psychology, Mount Allison University, Sackville, NB Canada; 4 Department of Psychology, Carleton University, Ottawa, ON Canada; 5 Westview Primary Care Network, Spruce Grove, AB Canada; 6 Department of Psychiatry, University of British Columbia, Vancouver, BC Canada; 7 Department of Paediatrics, University of Calgary, Calgary, AB Canada; 8 Faculty of Kinesiology, Sport, and Recreation, University of Alberta, Edmonton, AB Canada; 9 Patient and Family Partner, Edmonton, AB Canada; 10 Department of Health Research Methods, Evidence, and Impact, McMaster University, Hamilton, ON Canada; 11 Department of Pediatrics, University of Toronto, Toronto, ON Canada

**Keywords:** Attrition, Canada, Chronic Disease Management, Feasibility study, Obesity, Pediatric, Randomized trial

## Abstract

**Background:**

Pediatric obesity management can be successful, but some families discontinue care prematurely (i.e., attrition), limiting treatment impact. Attrition is often a consequence of barriers and constraints that limit families’ access to obesity management. Family Navigation (FN) can improve access, satisfaction with care, and treatment outcomes in diverse areas of healthcare. To help our team prepare for a future effectiveness trial, the objectives of our randomized feasibility study are to (i) explore children’s and caregivers’ acceptability of FN and (ii) examine attrition, measures of study rigor and conduct, and responses to FN + Usual Care vs Usual Care by collecting clinical, health services, and health economic data.

**Methods:**

In our 2.5-year study, 108 6–17-year-olds with obesity and their caregivers will be randomized (1:1) to FN + Usual Care or Usual Care after they enroll in obesity management clinics in Calgary and Mississauga, Canada. Our Stakeholder Steering Committee and research team will use Experience-Based Co-Design to design and refine our FN intervention to reduce families’ barriers to care, maximizing the intervention dose families receive. FN will be delivered by a navigator at each site who will use logistical and relational strategies to enhance access to care, supplementing obesity management. Usual Care will be offered similarly at both clinics, adhering to expert guidelines. At enrollment, families will complete a multidisciplinary assessment, then meet regularly with a multidisciplinary team of clinicians for obesity management. Over 12 months, both FN and Usual Care will be delivered virtually and/or in-person, pandemic permitting. Data will be collected at 0, 3, 6, and 12 months post-baseline. We will explore child and caregiver perceptions of FN acceptability as well as evaluate attrition, recruitment, enrolment, randomization, and protocol integrity against pre-set success thresholds. Data on clinical, health services, and health economic outcomes will be collected using established protocols. Qualitative data analysis will apply thematic analysis; quantitative data analysis will be descriptive.

**Discussion:**

Our trial will assess the feasibility of FN to address attrition in managing pediatric obesity. Study data will inform a future effectiveness trial, which will be designed to test whether FN reduces attrition.

**Trial registration:**

This trial was registered prospectively at ClinicalTrials.gov (#NCT05403658; first posted: June 3, 2022).

**Supplementary Information:**

The online version contains supplementary material available at 10.1186/s40814-023-01246-w.

## Background

Pediatric obesity is prevalent [1], persistent [2, 3], and complex [4, 5]. Without intervention, obesity and its consequences usually track into adulthood [6–8], a pattern that entrenches with increasing obesity severity [9] and underscores the value of accessible and effective interventions. However, successful obesity management (i.e., reducing or stabilizing weight gain; improving obesity-related consequences) is challenging. Multidisciplinary interventions centered on family lifestyle and behavior changes can help children to manage their obesity [10, 11]. Success in managing obesity is typically achieved by adhering to treatment regimens and regularly attending clinical appointments [12]. Effective lifestyle and behavioral interventions require a moderate to high intervention dose (i.e., > 25 h of clinic contact over 6–12 months [13]) to optimize health benefits [14, 15]. Achieving this dose is difficult for many families due to barriers and constraints to accessing care, such as schedule availability, transportation costs, and variable motivation [16, 17]. Even the best obesity management interventions are undermined when families discontinue care prematurely [18].

In managing pediatric obesity, we aim to minimize attrition (i.e., permanently discontinue care [19]) so children and families benefit from care. Attrition levels are as high as 80% [20]; 30–40% attrition is common [21–24]. Attrition wastes healthcare resources, discourages families from accessing services in the future [25, 26], and exacerbates health inequities for families with limited resources [27]. High attrition in pediatric obesity management was first reported as a problem > 50 years ago [28], but experimental research is lacking given the magnitude of the problem, highlighting the need to create and test interventions to improve access, reduce attrition, and manage obesity successfully [29].

Despite the lack of experimental research, descriptive research has produced some insights about attrition. For example, a review [16] published by our team members revealed higher attrition in children ≥ 12 years old and among families receiving social assistance. Reasons for attrition included logistical barriers and unmet family needs or expectations. Often, attrition involves multiple factors [21, 26, 30], with a series of missed or cancelled appointments preceding attrition, reducing the potential for positive treatment outcomes. Conversely, families continue attending appointments for several reasons, including anticipated and actual treatment benefits and high-quality care [31]. Ongoing attendance can be facilitated by flexible family schedules, choice of clinic appointment times, adequate family resources, and high motivation in children [31]. Some families can reduce barriers and eliminate constraints that limit access to obesity management, but many lack social support and financial resources, so could benefit greatly from enhanced care.

Several strategies have the potential to reduce attrition. Along with using multiple modes of communication with patents and families [32], clinical researchers recommend combining strategies to enhance clinic appointment attendance [33], including reducing wait times, making reminder calls [34], and applying techniques such as motivational interviewing to reduce missed or cancelled appointments [35]. Missed appointments are lost opportunities, so clinicians who establish collaborative relationships with patients can enhance clinic attendance and adherence to therapy plans [36]. In a systematic review of longitudinal studies designed to reduce attrition in adults [37], a greater number of strategies was inversely related to attrition; however, of the 88 studies in this review, only 4 were randomized controlled trials (RCTs), underscoring the need for experimental research to test multiple strategies for reducing attrition. Telephone and electronic reminder systems that provided detailed information beyond basic appointment details, including resource sharing and rapport building, are effective in reducing attrition [38–41]. In adult obesity interventions, multi-component approaches, financial incentives, and self-monitoring decreased attrition [42]. Notably, none of the trials in this review were developed a priori to reduce attrition; they also neglected to include stakeholders as intervention co-designers, highlighting the need for well-designed trials that include families, clinicians, and researchers as partners.

People living with a chronic illness like obesity often have difficulty navigating the complex, fragmented healthcare system [43]. Such experiences catalyzed research on the impact of support workers known as patient or family navigators. Navigators have diverse backgrounds but similar roles—to help patients and families coordinate care [44], access community services [31], attend clinical appointments [45], overcome communication and information barriers [46], and receive social support and education [47]. These activities can transform healthcare delivery by improving access, satisfaction, and outcomes [43, 48, 49]. Family Navigation (FN) is a multifaceted care model delivered by navigators who explore barriers to accessing clinical care and help patients and families by facilitating self-management and access to care. Rather than providing clinical care, FN addresses barriers and provides social support [43]. Navigators advocate for, educate, and assist families with tailored support and access to resources within and beyond the healthcare system [43]. In adults, navigation improves clinic appointment attendance [50], treatment outcomes [43], and satisfaction with care [51], especially when patients and families require services across settings and sectors [52]. FN is a direct response to recommendations [53] for tailored support to families in pediatric obesity management. FN is novel in pediatrics [54], and evaluations are rare, underscoring the need for robust evidence on the effectiveness of this intervention [55].

Our goal is to conduct a feasibility study to prepare our team for a definitive RCT in which we will test the effectiveness of FN to reduce attrition in 6–17-year-olds in pediatric obesity management. To help our team prepare for this future RCT, the objectives of our randomized feasibility study are to (i) explore children’s and caregivers’ acceptability of FN and (ii) examine attrition, measures of study rigor and conduct, and responses to FN + Usual Care vs Usual Care using clinical, health services, and health economic data.

## Methods

### Study design

Our 2.5-year, multi-method, multi-center randomized feasibility study has two arms and parallel assignment. Our protocol follows the Consolidated Standards of Reporting Trials (CONSORT) extension for randomized pilot and feasibility trials [56] and the Standard Protocol Items: Recommendations for Interventional Trials (SPIRIT). Please see Table 1 for our SPIRIT checklist [57].

**Table 1:**
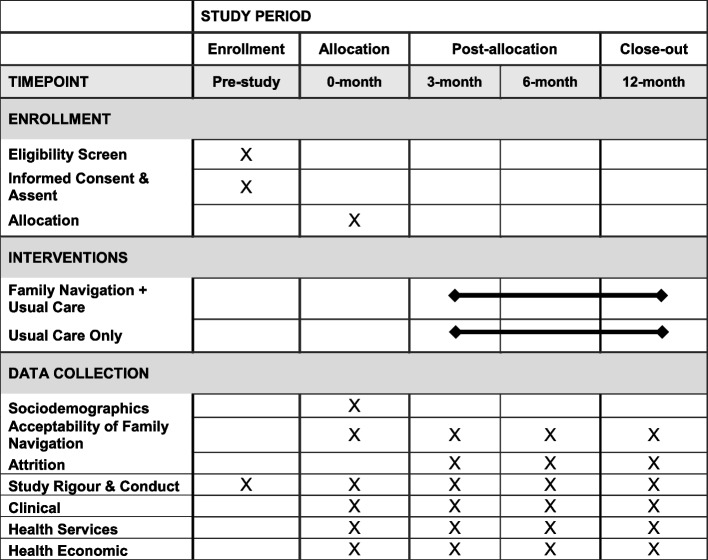
Schedule of enrollment, interventions, and data collection

### Study setting

Our study will be conducted at two clinical sites in Canada (Alberta Children’s Hospital in Calgary, AB; Trillium Health Partners in Mississauga, ON). Children will be assigned to an experimental (FN + Usual Care) or control (Usual Care) group after they enroll in publicly funded, multidisciplinary, pediatric obesity management clinics at our two sites.

### Stakeholder collaboration

Stakeholders have participated in several areas of our research. First, our application for research funding included both one caregiver and one colleague who works as a “community connector”; they played key roles in designing our study and conceptualizing our FN intervention. Second, our Stakeholder Steering Committee (SSC) is co-chaired by a (i) caregiver with experience in providing lived experience to academic teams through research studies and developing clinical practice guidelines and (ii) academic researcher with expertise in health, stress, and coping as well as navigation and peer-to-peer support for families of children with complex care needs. The SSC also includes other caregivers, older children, health care managers, clinicians, and research team members, who will meet regularly (e.g., monthly by videoconference; ad hoc by email) throughout our study.

As recommended [58], our SSC will work in partnership through several iterative stages, including the following tasks and outcomes: (i) engage in sharing experiences, perceptions, and preferences on access and attrition in managing pediatric obesity; (ii) discuss and evaluate existing navigation models with relevance to pediatric obesity; (iii) review FN design, theory, and implementation components, including logistical and relational strategies to increase access and reduce attrition; (iv) review and select outcome measures and data collection tools; (v) participate in qualitative and quantitative data interpretation to inform FN refinements and contextualize findings relevant to children, families, and clinicians; and (vi) contribute to knowledge translation products. Non-academic SCC members will receive $25 (CAN) gift cards for participating in each meeting. Finally, we consulted with several stakeholders who work with children and families, including social workers, school support workers, to ensure our FN intervention addressed the perceived needs of families who often access services to support child and caregiver health and well-being.

### Sample size

We will recruit 108 participants (54 per group and per site). Feasibility studies do not include formal sample size calculations [56], but experts recommend 12–36 per arm [59, 60]. We will enroll 27 participants per arm per site. This sample will give a 95% CI of width ≤ 0.34 for the difference in group proportions, assuming attrition is ≤ 0.4 in the control group. A sample size of 54 per arm will also allow 95% CIs of width ≤ 0.29 for other outcomes (e.g., proportion recruited). The sample size calculation for our future definitive RCT will be based on data from this feasibility study.

### Participant inclusion and exclusion criteria

At clinic enrolment, eligible participants must (i) be 6–17 years old, (ii) have a BMI ≥ 97th percentile [61], and (iii) have a primary caregiver (parent/guardian) agree to participate. Children of any sex or gender are eligible. Participants will be excluded if caregivers cannot communicate in English (< 5% of families in our clinics) since FN will be available in English only.

### Recruitment and enrollment

Research staff will work with clinical teams to offer families details about our study at clinic enrollment. Clinical team members will ask families if they are interested in learning about research at our clinics (i.e., consent to contact). If families respond “yes,” a research coordinator (RC) will contact caregivers to provide additional details about our study to enable enrollment. This process mirrors our approach in past multi-center studies at our two sites, including the Canadian Pediatric Weight management Registry (CANPWR; [62]). As an observational study, CANPWR offered no direct benefit to families, yet we recruited 66% (*n* = 1320/1992) of all children approached. We expect families to have a greater interest in this study because they have the potential to benefit directly from study participation via FN. Our annual volume of new referrals (Calgary: *n* ~ 300; Mississauga: *n* ~ 240) makes our study highly feasible. Recruitment will span ~ 12 months.

### Randomization and blinding

Our team biostatistician will computer-generate a permuted-block randomization sequence using child age on the day of consent (6–9 years, 10–13 years, 14–17 years) and clinic (Edmonton, Calgary, Mississauga) as subgroups to achieve balance across ages and clinics. The randomization sequence will be uploaded to Research Electronic Data Capture (REDCap) for centralized online randomization. Research coordinators (RCs) will then enter participant details into REDCap and manage randomization at each site.

We will conduct our study to reduce the risk of errors, follow best practices for real-world trials [63], and apply pediatric-specific recommendations to minimize the risk of bias [64]. To minimize selection bias, our online allocation process will be managed by team members not delivering interventions. RCs will collect study data to minimize response bias. Data analyses will be led by team members with no family contact. Our team biostatistician will create the randomization sequence but be blind to group assignment to minimize detection bias. RCs, navigators, clinicians, and families will know group assignment. Virtual appointments, which will remain the most common appointment type throughout our study, will limit interactions between families, minimizing ascertainment bias.

### Trial interventions

Our experimental group will receive FN + Usual Care. FN will be co-designed by our SSC and aims to reduce attrition by managing barriers and eliminating constraints that limit access to care. FN will increase access to a moderate to high intervention dose, increasing success in managing pediatric obesity. Although the initial design has started, refinements to the FN intervention (e.g., enhance navigator training/support) will continue to be made with our SSC, informed by study data and experience. Our control group will receive Usual Care only.

### Family navigation

The FN intervention will be co-designed with our SSC, including both theoretical and practical elements. Through co-design, we will maximize child- and family-centeredness, pragmatism, and intervention relevance by co-creating health services *with* and *for* children, caregivers, and clinicians [65].

Our participatory approach draws on key elements of Experience-Based Co-Design (EBCD) [66], an orientation that focuses on understanding SSC members’ experiences with health services, identifying potential improvements, and making changes together. With co-design, we increase intervention relevance and appropriateness, bringing together people who possess experiential knowledge (children, caregivers) with people who have expert knowledge (clinicians, researchers); the two knowledge systems enrich each other. Our team members have substantial experience in co-designing and refining health services and interventions in partnership with stakeholders [67–71].

Our two clinics will have a navigator to deliver FN. Navigators will be trained and equipped with resources for individualized support to benefit families in managing pediatric obesity. Navigators will start by orienting families to the intervention and completing a detailed needs assessment, highlighting areas for support to manage barriers and remove constraints to accessing pediatric obesity management. Strategies used by the navigator may include (i) communicating via text message to schedule appointments, providing appointment reminders, celebrating successes, exploring solutions for barriers to care, and sharing educational resources; (ii) having flexible navigator appointments (evenings, weekends, virtual); and (iii) providing parking/transit passes in-person visits of families with navigators, clinicians, and researchers.

Navigator appointments will supplement family appointments with clinicians for pediatric obesity management, increasing professional contact. Navigators will use principles of motivational interviewing (MI) [72] and focus on listening to and validating family challenges, exploring the desire to continue pediatric obesity management. They will foster a safe, non-judgmental space for families to discuss expectations and experiences as well as empower families to access resources and services that optimize care within and beyond the clinic. Navigators will be flexible and responsive. In some cases, they will work intensively with families, liaising regularly with clinicians to integrate care. In other cases, they will interact with families exclusively. Family preferences and needs drive appointment frequency with navigators: weekly, biweekly, or monthly; virtual (videoconference or phone) or in-person, pandemic permitting; 30–60 min long. As a tailored intervention, FN acknowledges that the desire to maximize adherence to pediatric obesity management varies by family and that ambivalence is common in managing obesity [30]. Navigator–family discussions may include children and caregivers together or separately, based on preferences and needs. Navigators will work with the research and clinical teams to adhere to a communication and documentation protocol if families disclose information (e.g., child safety) beyond their scope of practice. Navigators will hold an undergraduate degree in a relevant field (e.g., psychology, nursing, social work), complete advanced training in MI, and receive ongoing mentorship to maintain proficiency [73]. Registered psychologist team members (JG, AB) will oversee training and mentorship with a model that maximizes MI skill development and fidelity. Each navigator will complete ~ 80 h of MI training (e.g., readings, workshop) as recommended to maintain competence [74]. At the study start, all navigators will participate in a virtual workshop on strategies to mitigate obesity bias and stigma [75, 76], which will be led by a team member with expertise in this area (AA). In readings and discussions, navigators will gain perspective and expertise on children and families from diverse backgrounds and cultures. During the study, psychologist team members (JG, AB) will use the MI Supervision & Training Scale [77] to provide structured feedback to navigators on proficiency, maximizing fidelity to MI within and between navigators. After giving detailed feedback to navigators on their first several sessions with families, our team members will review randomly selected sessions throughout the study to highlight areas of strength and improvement for navigators. Navigators’ MI sessions with families will be recorded digitally and uploaded to a secure platform for data storage.

### Usual care

Our control intervention is Usual Care for managing pediatric obesity, delivered similarly across our two clinics by multidisciplinary teams who follow family-centered care principles [78] and guidelines [79]. At clinic presentation, children will complete a comprehensive health assessment to inform lifestyle (e.g., diet, physical activity, sedentary activity, sleep) and behavioral goal setting and subspecialty medical referrals, if indicated. Children and caregivers attend clinic visits regularly to make and maintain healthy changes. Appointments with physicians occur at least every 6 months, and more frequently with other clinicians (e.g., dietitian, psychologist). The dose and duration of pediatric obesity management vary according to family needs, motivation, and illness severity. Our two clinics offer 1-on-1 virtual care by videoconference or phone, with in-person visits restarting, pandemic permitting.

### Duration of treatment and follow-up

Intervention duration is 12 months for both experimental (FN + Usual Care) and control (Usual Care only) groups. Qualitative data will be collected at 3 intervals (0 [baseline], 3–6, and 12 months post-baseline). Quantitative data will be collected at 4 intervals (0 [baseline], 3, 6, and 12 months post-baseline).

### Data collection and management

To assess children’s and caregivers’ perceived acceptability of FN (objective 1), we will complete ~ 30-min, semi-structured, 1-on-1 interviews with families (see Additional file 1 for caregiver interview guide). We will group children into 6–9 years, 10–13 years, and 14–17 years. These groupings are based on potential developmental differences that could influence participants’ perceptions and experiences of the intervention. For the 6–9 years group, only caregivers will be interviewed as children of this age may have difficulty giving detailed insights on accessibility, and caregivers are responsible for virtually all decision-making for children in this age range. For 10–13 years and 14–17 years groups, children and caregivers will be interviewed independently, although child-caregiver (dyad) interviews will be offered (i.e., interviews will be completed as a family if children feel more comfortable with their caregiver present). We plan to interview participants in the experimental group (*n* = 54 caregivers; *n* = 36 10–17-year-olds) three times: before FN intervention delivery (0 month [baseline]), mid-intervention (3–6 months post-baseline), and at intervention completion (12 months post-baseline). However, the final sample size may be lower if (i) data saturation is achieved along the way and/or (ii) families discontinue care prematurely or terminate study participation. RCs at our two sites will conduct interviews virtually (videoconference, phone) or in-person, pandemic permitting. Interviews will be audio-recorded, transcribed verbatim, and managed with NVivo 11 (QSR, Australia).

To evaluate attrition and measures of study rigor and conduct (objective 2), we will compare outcomes against pre-set success indicators. We have a conceptual definition of attrition (permanently discontinue care [19]), but a universal, operational definition does not exist [16, 17]. For this reason, our operational definition will include three categories: “yes,” “no,” and “unknown,” which will be recorded identically for experimental (FN + Usual Care) and control (Usual Care only) groups. If a child discontinues pediatric obesity management at any point up to 12 months post-baseline or if an appointment is missed or cancelled without rescheduling and we have no follow-up communication with the family after four phone/text messages over 4 weeks and no scheduled upcoming appointments, they will be categorized as “yes.” If a child remains active in pediatric obesity management at 12 months post-baseline, they will be classified as “no.” If their status cannot be confirmed, they will be classified as “unknown.” The exact date of attrition will be pinpointed as a child’s last recorded interaction with a pediatric obesity management clinician or navigator, confirmed via primary (child’s medical record, clinic scheduling system) and secondary sources (families, clinicians, navigators [experimental group only]).

In addition, we will measure several secondary outcomes given their established or possible links to attrition [80–85], which will be collected for descriptive purposes and hypothesis generation. RCs will review children’s medical records to retrieve sociodemographic and clinical data, using standard case report forms and processes that adhere to recommendations for medical record reviews to optimize accuracy [86, 87]. For child- and caregiver-reported data, families will complete gender-neutral surveys created using REDCap that will be accessible virtually by desktop, tablet, or smart phone.

#### Clinical

Children’s weight (nearest 0.1 kg) and height (nearest 0.1 cm) will be collected to calculate BMI, BMI percentile, and BMI *z*-score [61]. Families will complete questionnaires on (i) health-related quality of life (HRQoL) assessed by child (self-report; 8–12 years and 13–18 years versions) and caregiver (proxy report) using the PedsQL 4.0 [88] and the Health Utility Index-3 (HUI-3) [89]; (ii) child- and caregiver-reported experienced and implicit weight-related stigma [90]; (iii) Working Alliance Inventory to quantify the strength of therapy relationship between both caregivers and navigators as well as caregivers and clinicians [91]; (iv) caregiver-reported treatment expectations for pediatric obesity management [83]; (v) child and caregiver motivation to change lifestyle and behavioral habits [92]; and (vi) caregiver-rated healthcare satisfaction using the PedsQL Healthcare Satisfaction Generic Module 3.0 [93]. RCs will document harms (i.e., adverse events ([AEs]), which may reveal unintended intervention effects. We will work with our SSC and Trial Steering Committee (TSC) to identify a list of potential harms related to managing pediatric obesity. Broader than adverse events, our list of potential harms will include unintended consequences experienced by families in FN + UC and UC groups.

#### Health services

RCs will track family health care use, including appointment (i) frequency (count); (ii) type (e.g., navigator, physician); (iii) mode (e.g., videoconference, in-person); (iv) duration (e.g., 15-min intervals); and (v) changes (e.g., cancelled, missed). Data will be retrieved from children’s medical records and electronic scheduling systems.

#### Health economics

We will collect descriptive data for future planning, estimating costs for FN intervention development (e.g., mentoring navigators), intervention delivery (e.g., session frequency), healthcare resource use (e.g., physician visits), and family-related time lost (e.g., travel). To inform future economic evaluations, we will use the HUI-3, which measures eight domains of HRQoL and generates a health utility score to calculate quality-adjusted life years (QALYs) associated with each intervention under an area under the curve approach.

#### Sociodemographic data

We will collect birth date, relationship between child and caregiver, ethnicity, and socioeconomic status by self-report from families on the case report form (based on Statistics Canada classifications). Children and caregivers will report their sex at birth [94] and gender [95] according to Statistics Canada definitions.

### Participant incentives

Families will be offered $25 (CAN) gift cards at each data collection time point.

### Data analyses

To inform any modifications before our definitive RCT, we will compare study data to pre-set feasibility criteria (Table 2). For objective 1 (measuring acceptability), we will perform a theoretically informed analysis of interview data using the Theoretical Framework of Acceptability (TFA; [96]). Our theoretically informed analysis situates the professional knowledge of the researcher, allows transparent examination of the research by the reader, and has two main characteristics: how data are structured and how data are interpreted [97]. The structure of our analytic process will be anchored in the theoretical framework. We will interpret the meaning of participants’ words vis-à-vis the theory while allowing new themes to develop. Our analysis will begin after the first interview and be ongoing during the study. RCs at each site will conduct initial coding, using TFA as a guide to structure themes while remaining open to identifying new themes, with line-by-line analysis. Initial findings will be shared with our SSC for review and discussion, then applied to the full data set. In analyzing the full data set, we will remain open to identifying new themes not accounted for in the TFA, reflecting our sensitive use of theory to guide analysis. For rigor and transparency, we will complete the Consolidated Criteria for Reporting Qualitative Research (COREQ) [98].

**Table 2:** Acceptability and study rigor and conduct data, with thresholds for assessing feasibility of future definitive randomized controlled trial

Methodological issues	Comments^a^	Feasibility data^a^	Criteria^b^	Feasible? (Y/N)	Need to modify pre-RCT? (Y/N)
1.Was the Family Navigation intervention acceptable to children and caregivers based on the 7 domains from the Theoretical Framework of Acceptability (TFA), including: a. Affective attitude b. Burden c. Perceived effectiveness d. Ethicality e. Intervention coherence f. Opportunity costs g. Self-efficacy	(e.g., data from 1-on-1 interviews)	(e.g., data will be analyzed using thematic analysis, which precludes quantification; 7 themes, plus additional sub-themes)	For each of the 7 domains, findings will be reviewed, discussed, and interpreted with our Stakeholder Committee and research team to determine whether parts of the Family Navigation intervention should remain unchanged or if changes are needed before implementing in our definitive RCT	(e.g., “yes” for all 7 domains)	(e.g., “yes” for 1 domain; “no” for 6 domains)
2.What proportion of participants approached were eligible?			≥ 90% approached were eligible		
3.Did recruited participants complete consent/assent procedures?			≥ 95% recruited participants completed consent/assent procedures		
4.Did recruitment lead to successful enrollment?			100% sample size goal achieved		
5.Did participants agree to randomization?			≥ 95% participants agreed to be randomized		
6.Did randomization yield equality across groups?			Equal numbers of participants randomized to experimental and control groups		
7.Were blinding procedures effective?			100% analysts remained blinded to group assignment		
8.Were outcome assessments completed?			≥ 95% participants retained in the study completed outcome assessments		
9.How complete were outcome assessments at all study measurement intervals?			≥ 95% outcome assessments were complete		
10. Were outcome assessments burdensome for families?			≥ 90% children and caregivers disagreed that outcome assessments were burdensome		
11. Was study protocol acceptable to children and caregivers?			≥ 90% children and caregivers agreed study protocol was acceptable		
12. Was the level of attrition adequate within experimental (Family Navigation + Usual Care) and control (Usual Care only) groups at 12 months?			Experimental (FN + UC): 15–25% attrition Control (UC): 30–40% attrition		
13. Was collection of attrition data adequate to calculate sample size for definitive RCT?			≥ 95% participants had attrition data collected by 12 months post-baseline		
14. Were logistics of running a multi-center trial assessed?			Review procedures (ongoing and end-of-grant) with investigators, research staff, navigators		
15. Did all components of the protocol work together adequately?			Review procedures (ongoing and end-of-grant) with investigators, research staff, navigators		

^a^Comments and Feasibility Data columns will be populated with study data collected during our study; example provided for context

^b^Criteria column thresholds based on objective criteria (when possible) and experience gained through study implementation as well as data analysis and interpretation with Stakeholder Committee and research team

For objective 2 (measuring attrition; study rigor and conduct; clinical, health services, economic outcomes), we will describe continuous data by summaries (means, medians, ranges) and categorical variables with frequency distributions. Data will be described for each group (e.g., sex, gender), stratification (e.g., age [6–9 years, 10–13 years, 14–17 years]), and clinic (Calgary, Mississauga). Group differences in outcomes will be calculated with 95% CIs. To complete power calculations for our definitive RCT, the 95% CI for the primary outcome (attrition yes/no at 12 months post-baseline) will be used. *R* [99] will be used for statistical analysis by a data analyst blinded to group assignments. Our SSC will discuss and contextualize study findings. REDCap will house quantitative data and generate data files for analyses. Consistent with feasibility studies [56], our analyses will be descriptive. Uncertainty exists on what elements(s) of FN are essential to optimize intervention effects [43]. In response, the participatory nature of our research, inclusion of qualitative and quantitative data sources, and heavy participation of our SSC will provide a full assessment of FN, revealing vital insights into how our experimental intervention can optimize treatment impact for managing pediatric obesity in our future definitive RCT.

Qualitative data analysis will occur throughout the study. Quantitative data analysis will occur at study completion only. Based on literature reviews of attrition and pediatric obesity management [16, 17], subgroup analyses will be exploratory and descriptive. We will describe attrition and other outcomes (e.g., HRQoL, intervention dose received) in experimental and control groups to explore potential differences by age, sex, gender, clinic, and changes within and between these subgroups over time.

### Ethical considerations

Children and caregivers can have emotional responses when discussing obesity-related issues. Our study takes place in multidisciplinary clinics, which include mental health professionals. Our study leaders (GDCB, JH, IZ) will work with research staff, navigators, clinicians, and administrative staff at our clinics to develop clear and specific processes to ensure children and caregivers are able to access mental health support (within or beyond our clinics, if relevant) when support is needed.

## Discussion

Attrition in managing pediatric obesity is a common occurrence. Families who attend more intervention sessions for obesity management and remain enrolled in care for longer achieve the greatest health improvements [100–103], observations that highlight the value of our study. We anticipate our findings will provide evidence that attrition has the potential to be reduced. The heterogeneity of approaches tested, small number of studies, sub-optimal study quality, and variable responses highlight the imperative for experimental research like ours to test evidence-based, theory-informed strategies such as FN that may reduce attrition in managing pediatric obesity. Our feasibility study represents a key next step in addressing obesity in children to help families get the most out of their care and optimizing the use of valuable health care resources.

### Knowledge translation

Our knowledge translation (KT) plan includes a blend of integrated and end-of-grant activities, which were informed using an established framework [104]. Children, caregivers, and clinicians from our SSC will partner with study leaders to co-author summaries of study results for our target audiences, providing real-world context for our findings and emphasizing key messages in plain language. Our integrated KT includes purposeful activities that are essential for study success, including formal meetings for our SSC and TSC, daily communication between research staff and clinicians, qualitative data analysis and interpretation that spans our project, and regular email correspondence to update stakeholders. Several of our team members lead multidisciplinary clinics for managing pediatric obesity, so study-related discussions and decisions will influence how our clinics plan and offer health services. We also lead provincial health system networks, with direct communication lines to enable province-level dissemination to clinicians and decision makers. Team members have established relationships with colleagues from Obesity Canada. We will share study data with public and professional audiences through newsletters and social media in the Obesity Canada community (> 50,000 members), with reach beyond academia and healthcare. At study completion, team members will work with public affairs experts at Obesity Canada and their respective institutions on press releases about findings. Results will be applied directly to inform our future definitive RCT to reduce attrition in pediatric obesity management.

### Trial oversight

Our TSC will meet at the start of our study and annually thereafter. TSC members include three arms-length, national research experts with backgrounds in pediatrics, obesity, clinical trials, and qualitative research who will form the committee with our three study leaders (GDCB, JH, IZ). Our TSC will review the study progress, approve the protocol and any amendments, and resolve any emerging challenges. A Data Safety and Monitoring Committee will not be established because the UAlberta Human Research Ethics Board views our study as a low-risk intervention.

### Trial status

Study activities were delayed due to the COVID-19 pandemic. Funding was received in July, 2021, preparatory study activities began in September, 2021, and participant recruitment began in October, 2022.

## Supplementary Information

**Additional file 1: Supplementary File 1.** Perceived Acceptability of Family Navigation Intervention – Caregiver Interview Guide (DRAFT).

## Data Availability

The datasets generated and/or analyzed during the current study are not publicly available since participants will complete informed and written consent (or assent) procedures explaining that study data are confidential and will only be shared within our research team. Summary data are available from the corresponding author upon reasonable request.

## Abbreviations

AE: Adverse event
BMI: Body mass index
CANPWR: CANadian Pediatric Weight management Registry
CI: Confidence interval
CIHR: Canadian Institutes of Health Research
CONSORT: Consolidated Standards of Reporting Trials
EBCD: Experience-Based Co-Design
FN: Family Navigation
HRQoL: Health-related quality of life
HUI: Health Utility Index
KT: Knowledge translation
MI: Motivational interviewing
QALY: Quality-adjusted life years
RC: Research Coordinator
RCT: Randomized controlled trial
SPIRIT: Standard Protocol Items: Recommendations for Interventional Trials
SSC: Stakeholder Steering Committee
TFA: Theoretical Framework of Acceptability
TSC: Trial Steering Committee

## References

[1] Rodd C, Sharma AK (2016). Recent trends in the prevalence of overweight and obesity among Canadian children. CMAJ.

[2] Simmonds M, Burch J, Llewellyn A, Griffiths C, Yang H, Owen C, Duffy S, Woolacott N (2015). The use of measures of obesity in childhood for predicting obesity and the development of obesity-related diseases in adulthood: a systematic review and meta-analysis. Health Technol Assess.

[3] Lo JC, Chandra M, Sinaiko A, Daniels SR, Prineas RJ, Maring B, Parker ED, Sherwood NE, Daley MF, Kharbanda EO, Adams KF, Magid DJ, O'Connor PJ, Greenspan LC (2014). Severe obesity in children: prevalence, persistence and relation to hypertension. Int J Pediatr Endocrinol.

[4] Hadjiyannakis S, Ibrahim Q, Li J, Ball GDC, Buchholz A, Hamilton JK, Zenlea I, Ho J, Legault L, Laberge AM, Thabane L, Tremblay M, Morrison KM (2019). Obesity class versus the Edmonton Obesity Staging System for pediatrics to define health risk in childhood obesity: results from the CANPWR cross-sectional study. Lancet Child Adolesc Health.

[5] Avis JL, Bridger T, Buchholz A, Chanoine JP, Hadjiyannakis S, Hamilton J, Jetha MM, Legault L, Morrison KM, Wareham A, Ball GD (2014). It's like rocket science…only more complex: challenges and experiences related to managing pediatric obesity in Canada. Expert Rev Endocrinol Metab.

[6] Callo Quinte G, Barros F, Gigante DP, de Oliveira IO, Dos Santos Motta JV, Horta BL (2019). Overweight trajectory and cardio metabolic risk factors in young adults. BMC Pediatr.

[7] Cunningham SA, Datar A, Narayan KMV, Kramer MR (2017). Entrenched obesity in childhood: findings from a national cohort study. Ann Epidemiol.

[8] Schuster MA, Elliott MN, Bogart LM, Klein DJ, Feng JY, Wallander JL, Cuccaro P, Tortolero SR (2014). Changes in obesity between fifth and tenth grades: a longitudinal study in three metropolitan areas. Pediatrics.

[9] Woo JG, Zhang N, Fenchel M, Jacobs DR, Hu T, Urbina EM, Burns TL, Raitakari O, Steinberger J, Bazzano L, Prineas RJ, Jaquish C, Juonala M, Ryder JR, Daniels SR, Sinaiko A, Dwyer T, Venn A (2020). Prediction of adult class II/III obesity from childhood BMI: the i3C consortium. Int J Obes (Lond).

[10] Al-Khudairy L, Loveman E, Colquitt JL, Mead E, Johnson RE, Fraser H, Olajide J, Murphy M, Velho RM, O'Malley C, Azevedo LB, Ells LJ, Metzendorf MI, Rees K (2017). Diet, physical activity and behavioural interventions for the treatment of overweight or obese adolescents aged 12 to 17 years. Cochrane Database Syst Rev.

[11] Mead E, Brown T, Rees K, Azevedo LB, Whittaker V, Jones D, Olajide J, Mainardi GM, Corpeleijn E, O'Malley C, Beardsmore E, Al-Khudairy L, Baur L, Metzendorf MI, Demaio A, Ells LJ (2017). Diet, physical activity and behavioural interventions for the treatment of overweight or obese children from the age of 6 to 11 years. Cochrane Database Syst Rev.

[12] Boff RM, Liboni RPA, Batista IPA, de Souza LH, Oliveira MDS (2017). Weight loss interventions for overweight and obese adolescents: a systematic review. Eat Weight Disord.

[13] Wilfley DE, Staiano AE, Altman M, Lindros J, Lima A, Hassink SG, Dietz WH, Cook S (2017). Improving Access and Systems of Care for Evidence-Based Childhood Obesity Treatment Conference Workgroup. Improving access and systems of care for evidence-based childhood obesity treatment: conference key findings and next steps. Obesity (Silver Spring).

[14] Hoedjes M, Makkes S, Halberstadt J, Noordam H, Renders CM, Bosmans JE, van der Baan-Slootweg OH, Seidell JC (2018). Health-related quality of life in children and adolescents with severe obesity after intensive lifestyle treatment and at 1-year follow-up. Obes Facts.

[15] Reinehr T, Lass N, Toschke C, Rothermel J, Lanzinger S, Holl RW (2016). Which amount of BMI-SDS reduction is necessary to improve cardiovascular risk factors in overweight children?. J Clin Endocrinol Metab.

[16] Dhaliwal J, Nosworthy NM, Holt NL, Zwaigenbaum L, Avis JL, Rasquinha A, Ball GD (2014). Attrition and the management of pediatric obesity: an integrative review. Child Obes.

[17] Skelton JA, Beech BM (2011). Attrition in paediatric weight management: a review of the literature and new directions. Obes Rev.

[18] Hagman E, Danielsson P, Lindberg L, Marcus C, BORIS Steering Committee (2020). Paediatric obesity treatment during 14 years in Sweden: lessons from the Swedish Childhood Obesity Treatment Register-BORIS. Pediatr Obes.

[19] Baekeland F, Lundwall L (1975). Dropping out of treatment: a critical review. Psychol Bull.

[20] Dolinsky DH, Armstrong SC, Østbye T (2012). Predictors of attrition from a clinical pediatric obesity treatment program. Clin Pediatr.

[21] De Miguel-Etayo P, Muro C, Santabárbara J, López-Antón R, Morandé G, Martín-Matillas M, Azcona-San Julián MC, Martí A, Campoy C, Marcos A, Moreno LA, Garagorri JM, EVASYON Study Group (2016). Behavioral predictors of attrition in adolescents participating in a multidisciplinary obesity treatment program: EVASYON study. Int J Obes (Lond).

[22] Warschburger P, Kröller K (2016). Loss to follow-up in a randomized controlled trial study for pediatric weight management (EPOC). BMC Pediatr.

[23] Walker SE, Smolkin ME, O’Leary M, Cluett SB, Norwood VF, Deboer MD, Gurka MJ (2012). Predictors of retention and BMI loss or stabilization in obese youth enrolled in a weight loss intervention. Obes Res Clin Pract.

[24] Ball GDC, Mackenzie KA, Newton MS, Alloway CA, Slack JM, Plotnikoff RC, Goran MI (2011). One-on-one lifestyle coaching for managing adolescent obesity: experience from a real-world, clinical setting. Paediatr Child Health.

[25] Sallinen Gaffka BJ, Frank M, Hampl S, Santos M, Rhodes ET (2013). Parents and pediatric weight management attrition: experiences and recommendations. Child Obes.

[26] Skelton JA, Irby MB, Beech BM, Rhodes SD (2012). Attrition and family participation in obesity treatment programs: clinicians' perceptions. Acad Pediatr.

[27] Buscemi J, Blumstein L, Kong A, Stollery MR, Schiffer L, Odoms-Young A, Bittner C, Fiztgibbon ML (2015). Retaining traditionally hard to reach participants: lessons learned from three childhood obesity studies. Contemp Clin Trials.

[28] Massengale ON (1965). The obese adolescent. Observations on etiology, management, prevention. Clin Pediatr (Phila).

[29] Miller BML, Brennan L (2015). Measuring and reporting attrition from obesity treatment programs: a call to action!. Obes Res Clin Prac.

[30] Hampl S, Demeule M, Eneli I, Frank M, Hawkins MJ, Kirk S, Morris P, Sallinen BJ, Santos M, Ward WL, Rhodes E (2013). Parent perspectives on attrition from tertiary care pediatric weight management programs. Clin Pediatr (Phila).

[31] Loskutova NY, Tsai AG, Fisher EB, LaCruz DM, Cherrington AL, Harrington TM, Turner TJ, Pace WD (2016). Patient navigators connecting patients to community resources to improve diabetes outcomes. J Am Board Fam Med.

[32] Brueton V, Stenning SP, Stevenson F, Tierney J, Rait G (2017). Best practice guidance for the use of strategies to improve retention in randomized trials developed from two consensus workshops. J Clin Epidemiol.

[33] Molfenter T (2013). Reducing appointment no-shows: going from theory to practice. Subst Use Misuse.

[34] McLean SM, Booth A, Gee M, Salway S, Cobb M, Bhanbhro S, Nancarrow SA (2016). Appointment reminder systems are effective but not optimal: results of a systematic review and evidence synthesis employing realist principles. Patient Prefer Adherence.

[35] Chiappetta L, Stark S, Mahmoud KF, Bahnsen KR, Mitchell AM (2018). Motivational interviewing to increase outpatient attendance for adolescent psychiatric patients. J Psychosoc Nurs Ment Health Serv.

[36] Watt BD, Dadds MR (2007). Facilitating treatment attendance in child and adolescent mental health services: a community study. Clin Child Psychol Psychiatry.

[37] Robinson KA, Dinglas VD, Sukrithan V, Yalamanchilli R, Mendez-Tellez PA, Dennison-Himmelfarb C, Needham DM (2015). Updated systematic review identifies substantial number of retention strategies: using more strategies retains more study participants. J Clin Epidemiol.

[38] Boksmati N, Butler-Henderson K, Anderson K, Sahama T (2016). The effectiveness of SMS reminders on appointment attendance: a meta-analysis. J Med Syst.

[39] Nuti L, Turkcan A, Lawley MA, Zhang L, Sands L, McComb S (2015). The impact of interventions on appointment and clinical outcomes for individuals with diabetes: a systematic review. BMC Health Serv Res.

[40] Gurol-Urganci I, de Jongh T, Vodopivec-Jamsek V, Atun R, Car J (2013). Mobile phone messaging reminders for attendance at healthcare appointments. Cochrane Database Syst Rev.

[41] Warner ET, Glasgow RE, Emmons KM, Bennett GG, Askew S, Rosner B, Colditz GA (2013). Recruitment and retention of participants in a pragmatic randomized intervention trial at three community health clinics: results and lessons learned. BMC Public Health.

[42] Pirotta S, Joham A, Hochberg L, Moran L, Lim S, Hindle A, Brennan L (2019). Strategies to reduce attrition in weight loss interventions: a systematic review and meta-analysis. Obes Rev.

[43] McBrien KA, Ivers N, Barnieh L, Bailey JJ, Lorenzetti DL, Nicholas D, Tonelli M, Hemmelgarn B, Lewanczuk R, Edwards A, Braun T, Manns B (2018). Patient navigators for people with chronic disease: a systematic review. PLoS ONE.

[44] Salmond S, Echevarria M (2017). Healthcare transformation and changing roles for nursing. Orthop Nurs.

[45] Allemang B, Allan K, Johnson C, Cheong M, Cheung P, Odame I, Ward R, Williams S, Mukerji G, Kuo KHM (2019). Impact of a transition program with navigator on loss to follow-up, medication adherence, and appointment attendance in hemoglobinopathies. Pediatr Blood Cancer.

[46] Freeman HP (2012). The origin, evolution, and principles of patient navigation. Cancer Epidemiol Biomarkers Prev.

[47] Knierim SD, Moore SL, Raghunath SG, Yun L, Boles RE, Davidson AJ (2018). Home visitations for delivering an early childhood obesity intervention in Denver: parent and patient navigator perspectives. Matern Child Health J.

[48] Peart A, Lewis V, Brown T, Russell G (2018). Patient navigators facilitating access to primary care: a scoping review. BMJ Open.

[49] Wells KJ, Campbell K, Kumar A, Clark T, Jean-Pierre P (2018). Effects of patient navigation on satisfaction with cancer care: a systematic review and meta-analysis. Support Care Cancer.

[50] Luckett R, Pena N, Vitonis A, Bernstein MR, Feldman S (2015). Effect of patient navigator program on no-show rates at an academic referral colposcopy clinic. J Womens Health.

[51] Hermann EA, Ashburner JM, Atlas SJ, Chang Y, Percac-Lima S (2018). Satisfaction with health care among patients navigated for preventive cancer screening. J Patient Exp.

[52] Azar R, Doucet S, Horsman AR, Charlton P, Luke A, Nagel DA, Hyndman N, Montelpare WJ (2020). A concept analysis of children with complex health conditions: implications for research and practice. BMC Pediatr.

[53] Tremblay M, Perez A, Rasquinha A, Avis JLS, Morrison KM, Chanoine JP, Legault L, Holt NL, Gokiert R, Sharma AM, Ball GDC (2016). Parents’ recommendations for improving the quality of health services for managing pediatric obesity in Canada. Acad Pediatr.

[54] Luke A, Doucet S, Azar R (2018). Paediatric patient navigation models of care in Canada: an environmental scan. Paediatr Child Health.

[55] Rollins M, Milone F, Suleman S, Vojvoda D, Sgro M, Barozzino T (2019). Patient navigators: mapping the route toward accessibility in health care. Paediatr Child Health.

[56] Eldridge SM, Chan CL, Campbell MJ, Bond CM, Hopewell S, Thabane L, Lancaster GA, PAFS consensus group (2016). CONSORT 2010 statement: extension to randomised pilot and feasibility trials. BMJ.

[57] Chan AW, Tetzlaff JM, Gøtzsche PC, Altman DG, Mann H, Berlin JA, Dickersin K, Hróbjartsson A, Schulz KF, Parulekar WR, Krleza-Jeric K, Laupacis A, Moher D (2013). SPIRIT 2013 explanation and elaboration: guidance for protocols of clinical trials. BMJ.

[58] Richard J, Azar R, Doucet S, Luke A (2020). Pediatric patient and family advisory councils: a guide to their development and ongoing implementation. J Patient Experience.

[59] Billingham SA, Whitehead AL, Julious SA (2013). An audit of sample sizes for pilot and feasibility trials being undertaken in the United Kingdom registered in the United Kingdom Clinical Research Network database. BMC Med Res Methodol.

[60] Julious SA (2005). Sample size of 12 per group rule of thumb for a pilot study. Pharm Stat.

[61] Secker D, Dietitians of Canada, Canadian Paediatric Society, College of Family Physicians of Canada, Community Health Nurses of Canada (2010). Promoting optimal monitoring of child growth in Canada: using the new WHO growth charts. Can J Diet Pract Res.

[62] Morrison KM, Damanhoury S, Buchholz A, Chanoine JP, Lambert M, Tremblay MS, Berall G, Hamilton J, Laberge AM, Legault L, Thabane L, Jakymyshyn M, Ambler KA, Ball GD (2014). The CANadian Pediatric Weight management Registry (CANPWR): study protocol. BMC Pediatr.

[63] Gluud LL (2006). Bias in clinical intervention research. Am J Epidemiol.

[64] Hartling L, Hamm M, Klassen T, Chan AW, Meremikwu M, Moyer V, Scott S, Moher D, Offringa M, StaR Child Health Group (2012). Standard 2: containing risk of bias. Pediatrics.

[65] Slattery P, Saeri AK, Bragge P (2020). Research co-design in health: a rapid overview of reviews. Health Res Policy Syst.

[66] Green T, Bonner A, Teleni L, Bradford N, Purtell L, Douglas C, Yates P, MacAndrew M, Dao HY, Chan RJ (2020). Use and reporting of experience-based codesign studies in the healthcare setting: a systematic review. BMJ Qual Saf.

[67] Mâsse LC, Vlaar J, Macdonald J, Bradbury J, Warshawski T, Buckler EJ, Hamilton J, Ho J, Buchholz A, Morrison KM, Ball GDC (2020). Aim2Be mHealth intervention for children with overweight and obesity: study protocol for a randomized controlled trial. Trials.

[68] Green J, Wills A, Mansfield E, Sur D, Zenlea IS (2019). Welcoming feedback: using family experience to design a pediatric weight management program. J Patient Exp.

[69] Ball GD, Mushquash AR, Keaschuk RA, Ambler KA, Newton AS (2017). Using intervention mapping to develop the Parents as Agents of Change (PAC©) intervention for managing pediatric obesity. BMC Res Notes.

[70] Avis JL, Holt NL, Maximova K, van Mierlo T, Fournier R, Padwal R, Cave AL, Martz P, Ball GD (2016). The development and refinement of an e-health screening, brief intervention, and referral to treatment for parents to prevent childhood obesity in primary care. Telemed J E Health.

[71] Ball GD, Farnesi BC, Newton AS, Holt NL, Geller J, Sharma AM, Johnson ST, Matteson CL, Finegood DT (2013). Join the conversation! The development and preliminary application of conversation cards in pediatric weight management. J Nutr Educ Behav.

[72] Rollnick S, Miller WR, Butler CC (2008). Motivational interviewing in health care: helping patients change behavior.

[73] Hall K, Staiger PK, Simpson A, Best D, Lubman DI (2016). After 30 years of dissemination, have we achieved sustained practice change in motivational interviewing?. Addiction.

[74] Schwalbe CS, Oh HY, Zweben A (2014). Sustaining motivational interviewing: a meta-analysis of training studies. Addiction.

[75] Alberga AS, Edache IY, Forhan M, Russell-Mayhew S (2019). Weight bias and health care utilization: a scoping review. Prim Health Care Res Dev.

[76] Alberga AS, Pickering BJ, Alix Hayden K, Ball GD, Edwards A, Jelinski S, Nutter S, Oddie S, Sharma AM, Russell-Mayhew S (2016). Weight bias reduction in health professionals: a systematic review. Clin Obes.

[77] Madson MB, Campbell T, Barrett DE, Brondino MJ, Melchert TP (2005). Development of the motivational interviewing supervision and training scale. Psychol Addict Behav.

[78] American Academy of Pediatrics (2003). Family-centered care and the pediatrician's role. Pediatrics.

[79] Lau DC, Douketis JD, Morrison KM, Hramiak IM, Sharma AM, Ur E, Obesity Canada Clinical Practice Guidelines Expert Panel (2007). 2006 Canadian clinical practice guidelines on the management and prevention of obesity in adults and children [summary]. CMAJ.

[80] McMaster CM, Gow ML, Neal R, Alexander S, Baur LA, Cohen J (2020). Acceptability of hospital-based pediatric weight management services among patients and families: a narrative synthesis. Child Obes.

[81] Brochu PM (2018). Weight stigma is a modifiable risk factor. J Adol Health.

[82] Pit-Ten Cate IM, Samouda H, Schierloh U, Jacobs J, Vervier JF, Stranges S, Lair ML, Beaufort C (2017). Can health indicators and psychosocial characteristics predict attrition in youths with overweight and obesity seeking ambulatory treatment? Data from a retrospective longitudinal study in a paediatric clinic in Luxembourg. BMJ Open.

[83] Rhodes ET, Boles RE, Chin K, Christison A, Testa EG, Guion K, Hawkins MJ, Petty CR, Sallinen Gaffka B, Santos M, Shaffer L, Tucker J, Hampl SE (2017). Expectations for treatment in pediatric weight management and relationship to attrition. Child Obes.

[84] Spence N, Newton AS, Keaschuk RA, Ambler KA, Jetha MM, Holt NL, Rosychuk RJ, Spence JC, Sharma AM, Ball GDC (2017). Predictors of short- and long-term attrition from the Parents as Agents of Change (PAC) randomized controlled trial for managing pediatric obesity. J Pediatr Health Care.

[85] De Miguel-Etayo P, Muro C, Santabárbara J, López-Antón R, Morandé G, Martín-Matillas M, Azcona-San Julián MC, Martí A, Campoy C, Marcos A, Moreno LA, Garagorri JM, EVASYON Study Group (2016). Behavioral predictors of attrition in adolescents participating in a multidisciplinary obesity treatment program: EVASYON study. Int J Obes.

[86] Sarkar S, Seshadri D (2014). Conducting record review studies in clinical practice. J Clin Diagn Res.

[87] Gearing RE, Mian IA, Barber J, Ickowicz A (2006). A methodology for conducting retrospective chart review research in child and adolescent psychiatry. J Can Acad Child Adolesc Psychiatry.

[88] Varni JW, Seid M, Kurtin PS (2001). PedsQL 4.0: reliability and validity of the Pediatric Quality of Life Inventory version 4.0 generic core scales in healthy and patient populations. Med Care.

[89] Horsman J, Furlong W, Feeny D, Torrance G (2003). The Health Utilities Index (HUI): concepts, measurement properties and applications. Health Qual Life Outcomes.

[90] Pearl RL, Puhl RM (2014). Measuring internalized weight attitudes across body weight categories: validation of the modified weight bias internalization scale. Body Image.

[91] Sturgiss EA, Sargent GM, Haesler E, Rieger E, Douglas K (2016). Therapeutic alliance and obesity management in primary care – a cross-sectional pilot using the working alliance inventory. Clin Obes.

[92] Sobell L, Sobell M. Motivational interviewing strategies and techniques: rationales and examples. 2008. www.nova.edu/gsc/forms/gsc-forms.html. Accessed 8 Oct 2020.

[93] Varni JW, Burwinkle TM, Dickinson P, Sherman SA, Dixon P, Ervice JA, Leyden PA, Sadler BL (2004). Evaluation of the built environment at a Children’s Convalescent Hospital: Development of the Pediatric Quality of Life Inventory™ parent and staff satisfaction measures for pediatric health care facilities. J Dev Behav Pediatr.

[94] Statistics Canada. Sex at birth of person. Government of Canada. www23.statcan.gc.ca/imdb/p3Var.pl?Function=DEC&Id=24101. Accessed 16 June 2022.

[95] Statistics Canada. Gender of person. Government of Canada. www23.statcan.gc.ca/imdb/p3Var.pl?Function=DECI&Id=1326692. Accessed 16 June 2022.

[96] Sekhon M, Cartwright M, Francis JJ (2017). Acceptability of healthcare interventions: an overview of reviews and development of a theoretical framework. BMC Health Serv Res.

[97] Hissa J, Timulak L (2020). Theoretically informed qualitative psychotherapy research: a primer. Couns Psychother Res.

[98] Tong A, Sainsbury P, Craig J (2007). Consolidated criteria for reporting qualitative research (COREQ): a 32-item checklist for interviews and focus groups. Int J Qual Health Care.

[99] R Core Team (2017). A language and environment for statistical computing.

[100] Nobles J, Griffiths C, Pringle A, Gately P (2017). Why consistent completion criterion are required in childhood weight management programmes. Public Health.

[101] Wilfley DE, Saelens BE, Stein RI, Best JR, Kolko RP, Schechtman KB, Wallendorf M, Welch RR, Perri MG, Epstein LH (2017). Dose, content, and mediators of family-based treatment for childhood obesity: a multisite randomized clinical trial. JAMA Pediatr.

[102] Theim KR, Sinton MM, Goldschmidt AB, Van Buren DJ, Doyle AC, Saelens BE, Stein RI, Epstein LH, Wilfley DE (2013). Adherence to behavioral targets and treatment attendance during a pediatric weight control trial. Obesity (Silver Spring).

[103] Kalarchian MA, Levine MD, Arslanian SA, Ewing LJ, Houck PR, Cheng Y, Ringham RM, Sheets CA, Marcus MD (2009). Family-based treatment of severe pediatric obesity: randomized, controlled trial. Pediatrics.

[104] Barwick M. (2008, 2013, 2019). Knowledge translation planning template. ON: The Hospital for Sick Children.

