# Supplementary material for: A multi-center, randomized, 12-month, parallel-group, feasibility study to assess the acceptability and preliminary impact of family navigation plus usual care versus usual care on attrition in managing pediatric obesity: a study protocol
Source: Pilot Feasibility Stud. 2023 Jan 23;9:14. doi: 10.1186/s40814-023-01246-w (PMC9868519; doi:10.1186/s40814-023-01246-w)
Supplement: Supplementary file 1 — Additional file 1: Supplementary File 1. Perceived Acceptability of Family Navigation Intervention – Caregiver Interview Guide (DRAFT). [file 40814_2023_1246_MOESM1_ESM.docx]

**Supplementary File 1. Perceived Acceptability of Family Navigation Intervention – Caregiver Interview Guide (DRAFT)**

**Preamble:**

*The purpose of this interview is to learn from our families (children and caregivers) about their perceptions, preferences, and experiences regarding our family navigation intervention. Specifically, this interview will explore the acceptability of our family navigation intervention. As a reminder, the purpose of family navigation is to help families improve access to care by addressing barriers that can reduce their ability to participate and engage in pediatric obesity management.*

*Through these interviews, our research team will learn about several issues related to acceptability as well as the strengths, limitations, and recommendations for improving our family navigation from you and families like you. Our results will inform improvements we make before we conduct a definitive randomized controlled trial to test the effectiveness of family navigation to reduce attrition in pediatric obesity management.*

**NOTE: The following questions are from the interview that will be completed at 3–6 months post-baseline, after participants have gained some experience with the Family Navigation intervention and pediatric obesity management.**

| **Categories** | **Questions and Probes** |
| --- | --- |
| ***Introduction*** | How long have you been attending the <insert name> clinic?  What has been your experience so far?  How many times have you spoken with <insert name>, the family navigator?  Have your discussions been by videoconference, phone, or in-person? |
| **Theoretical Framework of Acceptability** | |
| 1. ***Affective Attitude*** | Now that you’ve met with the navigator at the clinic a few times, how do you feel about her/him? Overall, how do you feel about the Family Navigation (FN) intervention? |
| 1. ***Burden*** | To date, how much effort has it been for you to participate in Family Navigation? How does that effort compared to the effort required for you to participate in obesity management? (*FN should make it easier for families to participate in the Usual Care for managing pediatric obesity. Follow-up questions can probe for examples, including if and how FN has made obesity management easier*). |
| 1. ***Perceived Effectiveness*** | To what degree do you feel that FN has achieved its intended purpose – to help you access services and supports for obesity management? (*Follow-up questions can prompt parents to share experiences and examples from within and beyond the clinic*). |
| 1. ***Ethicality*** | FN is intended to help you and your family (and families like yours) to access services and supports. How important is that approach to you? (*Query participants’ views of equity, diversity, and inclusiveness, including who they feel the FN intervention will help the most*). |
| 1. ***Intervention Coherence*** | Knowing what you know about FN, how do you feel about the intervention approach overall? (*Query their knowledge and understanding of FN and how it was designed to work*). |
| 1. ***Opportunity Costs*** | Have you (and your family) had to sacrifice or give up anything to participate in FN? In obesity management? In your view, what are the things you gave up (if any) to participate in the intervention? (*Query costs including time, financial, work, school, recreational activities, and whether the costs [real or perceived] were worthwhile*). |
| 1. ***Self-efficacy*** | How confident are you that you can perform the behaviour(s) required to participate in FN? Is your confidence the same or different when you consider the requirements for you to participate in obesity management? (*Follow-up to probe whether confidence is context specific, including different levels of confidence for different activities and commitments*). |
| **Open Questions** | |
| ***Logistical*** | Has your family experienced any barriers to attending clinical appointments (virtual or in-person)? *Probe for real and perceived barriers (e.g., time, other priorities, motivation).*  Has the navigator offered you any practical resources to help make it easier to access care at the clinic? *Probe for items among the bundle of resources (e.g., parking / transportation passes, text messaging, flexible appointments, resources beyond the clinic).*  What kinds of logistical (or practical) things have been most helpful for your family? Least helpful? *Probe to gain understanding about how and why.* |
| ***Relational*** | In the conversations you’ve had with the navigator, in general, what topics have you talked about? *Probe whether issues are related to or separate from obesity management.*  In general, do you meet with the navigator as a family or individually? *Probe for perceptions of their child’s experience with the navigator and the interactions between navigator-parent and navigator-child.*  What words would you use choose to describe the navigator? *Probe for a range of examples (e.g., good listener, rushed, sincere, distracted, available).*  Can you tell me about any times you (or your child) felt accepted (positively) or judged (negatively) by the navigator? *Probe for experiences and perceptions regarding obesity bias and stigma.* |
| ***Strengths & Limitations*** | In your opinion, what are the strengths of the family navigator intervention? *Probe for details and experiences.*  In your opinion, what are the limitations of the family navigator intervention? *Probe for details and experiences.* |
| ***Recommendations*** | Overall, do you have any recommendations for ways that we could improve the family navigation intervention?  Do you have any recommendations for practical resources that would make it easier for you or other families to access the clinic? Anything to add or remove? *Probe for details regarding logistical (e.g., financial incentives [including value], child-minding, weekend appointments).*  Do you have any recommendations for way to improve the inter-personal interactions you or other families have with the navigator? *Probe for details (e.g., interactions with clinicians, perceived training and support).* |
| ***Summary*** | Is there anything else you wish to add about your experiences and perceptions about the navigator (specifically) or the family navigation intervention (generally)? |

**FOOTNOTES:**

- The interview guide was based on the *Theoretical Framework of Acceptability* (Sekhon *et al., BMC Health Services Res.* 2017), which includes seven acceptability-related domains (affective attitude, burden, perceived effectiveness, ethicality, intervention coherence, opportunity costs, and self-efficacy). Several open questions were added to generate contextual data.
- Interviews will be conducted virtually (phone or videoconference) or in-person (pandemic permitting).
- Tailored guides will be created for interviews with both children (10–17 years old only) and caregivers. They will also be adapted based on study timing. For example, interviews conducted at baseline will include questions regarding *anticipated* intervention effectiveness while those held at study completion will include questions regarding *experienced* intervention effectiveness.
- Interview guides will be reviewed and refined by our Stakeholder Committee.
